# Unveiling and mapping the immune compartment in human Alzheimer’s disease

**DOI:** 10.1002/alz.085927

**Published:** 2025-01-03

**Authors:** Suzanne Buss, Kevin Couper, Michael Haley, Emily Brookes, Diego Gomez‐Nicola

**Affiliations:** ^1^ University of Southampton, Southampton United Kingdom; ^2^ University of Manchester, Manchester United Kingdom

## Abstract

**Background:**

The neuroimmune response, characterised by the proliferation and activation of microglial cells, is a driver of Alzheimer’s disease (AD). However, the extent of immune cell infiltration and interactions in the human AD brain are yet to be established in detail. While microglial cells are at the centre of this neuroimmune response, recent research has explored the possibility of peripheral immune cell involvement, typically focusing on T‐cell infiltration. There is currently a scarcity of studies using human tissues, with most of our understanding being derived from the study of animal models of AD‐like pathology, which lack the complexity and diversity of the human condition. Our study aims to advance our understanding of the complex interplay of immune cells and signals, helping to define how the neuroimmune response plays a role in driving AD pathogenesis.

**Methods:**

We used two complementary techniques that provide a comprehensive map of the immune compartment through providing data rich results and spatial context. Samples were derived from human fresh‐frozen and FFPE post‐mortem tissues at all Braak stages, obtained from UK brain banks. We used fluorescence activated nuclei sorting to isolate the key cellular populations in the brain, as well as other non‐resident immune cells. Single‐nuclei RNA sequencing is used to establish not only the microglial transcriptional diversity in AD but also what other immune populations we see residing in the AD brain. Imaging mass cytometry, a highly quantitative technique that utilises up to 40 metal conjugated antibodies simultaneously, has also been deployed to provide spatial context of the main microglial phenotypes and immune cell interactions via single‐cell bioinformatic analysis.

**Results:**

Preliminary data suggests that there are populations of peripheral immune cells infiltrating into the brain during AD which could be impacting disease outcome. Distinct microglial phenotypes have also been detected which reflect results of recent human single‐cell RNA sequencing studies. Future work includes increasing the sample number to further support our preliminary findings.

**Conclusion:**

By understanding the complex interplay of immune cells, not only with one another but with pathological and physical hallmarks, we could significantly advance our comprehension of AD pathogenesis and steer research into preventative and/or treatment options.

